# A Novel Unsupervised Adaptive Learning Method for Long-Term Electromyography (EMG) Pattern Recognition

**DOI:** 10.3390/s17061370

**Published:** 2017-06-13

**Authors:** Qi Huang, Dapeng Yang, Li Jiang, Huajie Zhang, Hong Liu, Kiyoshi Kotani

**Affiliations:** 1 State Key Laboratory of Robotics and System, School of Mechatronics Engineering, Harbin Institute of Technology, Harbin 150001, China; yangdapeng@hit.edu.cn (D.Y.); zhanghuajie001@163.com (H.Z.); hong.liu@hit.edu.cn (H.L.); 2 Research Center for Advanced Science and Technology, the University of Tokyo and PRESTO/JST, Tokyo 153-8904, Japan; kotani@neuron.t.u-tokyo.ac.jp

**Keywords:** long-term EMG pattern recognition, adaptive learning, concept drift, particle adaption, support vector classifier

## Abstract

Performance degradation will be caused by a variety of interfering factors for pattern recognition-based myoelectric control methods in the long term. This paper proposes an adaptive learning method with low computational cost to mitigate the effect in unsupervised adaptive learning scenarios. We presents a particle adaptive classifier (PAC), by constructing a particle adaptive learning strategy and universal incremental least square support vector classifier (LS-SVC). We compared PAC performance with incremental support vector classifier (ISVC) and non-adapting SVC (NSVC) in a long-term pattern recognition task in both unsupervised and supervised adaptive learning scenarios. Retraining time cost and recognition accuracy were compared by validating the classification performance on both simulated and realistic long-term EMG data. The classification results of realistic long-term EMG data showed that the PAC significantly decreased the performance degradation in unsupervised adaptive learning scenarios compared with NSVC (9.03% ± 2.23%, *p* < 0.05) and ISVC (13.38% ± 2.62%, *p* = 0.001), and reduced the retraining time cost compared with ISVC (2 ms per updating cycle vs. 50 ms per updating cycle).

## 1. Introduction

According to a survey on the usage of prostheses [1], 28% of the users are categorized as “prosthesis rejecters”, who use their prostheses no more than once a year, mainly because of the clumsy control of commercial prostheses. Compared with conventional control methods, the control scheme based on pattern recognition (PR), employing advanced feature extraction and classification technology, shows up the potential to leverage the intuitiveness and functionality of myoelectric control [2,3].

However, when employing PR-based methods to realize myoelectric control, interfering factors such as temperature and humidity changes, skin impedance variation, muscular fatigue, electrode shifting and limb position changes will cause classification degradation [4,5,6], hindering the clinical application and the commercialization of the PR-based EMG control scheme. After analyzing both industrial and academic demands, Farina et al. [7] divided the demand for reliability of upper limb prosthesis control system into two parts: (a) the robustness to instantaneous changes such as the electrode shifting when donning and doffing, and arm posture variation; and (b) the adaptability to slow changes such as muscular fatigue and skin impedance variation. The robustness is usually related to research on advanced signal recording methods, such as optimizing the size and the layout of EMG electrodes [8,9], employing high-density electrodes to get more information [10,11], and finding out invariant characteristics in EMG signals [12], whereas the adaptability is usually related to adaptive learning methods [13,14,15,16,17]. The objective of this paper is to employ the adaptive learning method based on the theory of concept drift to endow the PR classifier with adaptability to slow changes of EMG signals. Related work is described in the following section.

### 1.1. Related Work

#### 1.1.1. Adaptive Learning on Surface EMG Data

The phenomenon whereby the probability distribution of target data changes along time is called concept drift [18,19], where the term concept refers to the probability distribution in feature space. Hence, the adaptive learning is defined as the learning method that updates its predictive model online to track the concept drift. According to whether the learning method needs labeled samples to retrain the predictive model or not, the adaptive learning methods can be categorized into supervised adaptions [15,20,21] and unsupervised adaptions [16,22]. The supervised adaption is able to achieve high recognition accuracy, but at the cost of a cumbersome training process to acquire labeled samples repetitiously, whereas the unsupervised adaption is user-friendly but at the cost of reduced accuracy [13]. Therefore, the crux of the unsupervised adaption is to choose exemplars with high confidence. Researchers tried to evaluate the confidence of the classification for unlabeled samples by using the likelihood [14], the entropy [23], the consistency of classification decisions [24], or simply rejecting unknown data patterns [25]. However, those kinds of evaluations would introduce extra computational costs during the retraining processes and result in data jamming during online control. Specific to the predictive model, most researchers [13,14,16,20,21,22,25] chose linear discriminant analysis (LDA) because of its simplicity to retrain the model [26], whereas only a few researchers [27,28] chose support vector machine (SVM) [29,30], a better classifier that has solid mathematical foundation and good performance with small training sample set. The SVM has been used for non-adapting myoelectric control [31,32]. Designing adaptive classifier based on the SVM is challenging because: (a) the computational cost of the training process of SVM is unacceptable for real-time updating; (b) there is no direct mapping from the output of SVM decision function to the classification confidence [28], resulting in difficulties with choosing reliable exemplars. This paper is to design an adaptive classifier based on the SVM by overcoming those adverse conditions.

#### 1.1.2. Adaptive Learning Based on SVM

Based on standard SVM, two modifications were proposed to reduce the computational cost of the training process of the SVM: (a) incremental SVM (ISVM) [33], which reduced the computational cost by an incremental learning method, and (b) least square support vector machine (LS-SVM) [34], which reduced the computational cost by eliminating the heuristic nature from the training process of support vectors. Similar to the SVM, the incremental learning method could be applied on LS-SVM to further reduce the retraining cost [35,36]. To update the predictive model of LS-SVM with fewer labeled samples and lower computational cost, Tommasi et al. proposed a model adaption method [37], which could compute model coefficients from models prepared in advance. On the other hand, unsupervised adaptive learning methods based on the SVM suffered the accumulation of misclassification risk of unlabeled samples. Transductive learning method based on the SVM [38], which could relabel a sample after more samples being processed, was expected to suppress the accumulation of misclassification risk. However, the transductive learning method was computational costly and assumed the same probability distribution of source dataset and target dataset, which was inapplicable for adaptive learning. Another unsupervised adaptive learning method was to estimate the posterior probability of the output of SVM’s decision function and choose reliable samples accordingly [28]. The method was also computational costly when estimating the posterior probability repeatedly.

#### 1.1.3. Validation Method for Adaptive Learning

The major concern on validating the adaptive learning is how to build a picture of accuracy changes over time. The conventional cross validation method is inapplicable [17], because it assumes the same distribution is shared by the training data and the verification data. To the contrary, a commonly used method is computing prequential errors of sequential data, no matter the data were acquired from realistic environment or generated with controlled permutations. Unfortunately, specific to myoelectric control, the validation data sequences of most researches by now were limited to either four to six sessions continually acquired within 2 to 3 h [13,14,15,17,27], or numerous sessions acquired at two time points [22] (one for training, the other one for validating), which did not agree with the reality of using prostheses, and thus could not perfectly reflect the adaptability of the learning method. Some researchers argued that the closed-loop (or human-in-the-loop) learning and validation scheme could reveal the co-adaption between the user and the adaptive system [17]. However, the open-loop validating is essential to check the inherent adaptability of the adaptive learning method, and this paper will employ the open-loop validating method.

### 1.2. Significance of This Paper

According to the related work, we could conclude that the unsupervised adaptive learning issue remains an open problem. Existing methods based on LDA have the problem of high misclassification risk because of its unsophisticated predictive model, whereas methods based on SVM have the problem of low computation efficiency and the rapid accumulation of misclassification risk during updating. Particle adaptive classifier (PAC), proposed in this paper, is to address the problem of SVM-based unsupervised adaptive learning methods. It improves the computational efficiency by constructing universal incremental LS-SVM and representative-particles-based sparse LS-SVM. It reduces the accumulation of misclassification risk by employing neighborhood updating based on the principles of smoothness assumption and cluster assumption [39], the principles widely used in semi-supervised learning. To analyze the performance of adaptive learners, we design a validating paradigm that measures prequential errors of two kinds of data: (a) simulated data with controlled permutations, to analyze the adaptability of the adaptive learner, and (b) acquired data with the time span of one day, to check the actual applicability of the adaptive learner. To explore the feasibility in various working situations, we compared the performances of adaptive learners in both unsupervised and supervised retraining scenarios.

## 2. Materials and Methods

### 2.1. Principles of Adaptive Learning

Compared with non-adapting methods, adaptive learning methods are able to track the concept drift to compensate the long-term performance degradation, but introduce the retraining/updating risk. The retraining/updating risk is caused by adopting *wrong samples* to retrain/update the predictive model. The wrong samples refer to samples having labels conflicting with their ground truth labels according to current concept. In supervised adaptive learning scenarios, old samples are likely to be wrong samples because they may be outdated after concept drifting. In unsupervised adaptive learning scenarios, both outdated old samples and misclassified new samples are wrong samples, and such misclassification will result in the accumulation of the misclassification risk. Therefore, when designing an adaptive learning method, we should take the following facts into consideration:

(a) Since we don’t know when the concept drift happens, information from old data may on one hand be useful to the classification generalization, and on the other hand be harmful to the representability of the classifier [40]. Therefore, a good adaptive classifier should balance the benefit and the risk of using old data.
(b) Drifting concepts are learnable only when the rate or the extent of the drift is limited in particular ways [41]. In many studies, the extent of concept drift is defined as the probability that two concepts disagree on randomly drawn samples [19], whereas the rate of concept drift is defined as the extent of the drift between two adjacent updating processes [42]. Considering that the sample distribution and the recognition accuracy are immeasurable for a single sample, we define the dataset of samples with the same distribution as a *session*. In this way, *drift extent* (*DE*) is defined as the difference of sample distribution between two sessions, and *drift rate* (*DR*) is defined as the drift extent between two adjacent sessions in a validating session sequence.

### 2.2. Incremental SVC

#### 2.2.1. Nonadapting SVC

To solve the binary classification problem with a set of training pairs ${\left\{\left(x_{i},y_{i}\right)\right\}}_{i=1}^{l}$, $x_{i}\in R^{n}$, $y_{i}\in \{-1,+1\}$, a standard C-SVC [29] formulates the indicator function as $f(x)=\mathrm{sgn}(w^{T}\varphi (x)+b)$ and the optimization primal problem as follows:

(1) $$\begin{array}{l} \underset{w,b,\xi }{\min}\;J_{SVC}=\frac{1}{2}w^{T}w+C\sum_{i=1}^{l}\xi_{i}\\ s.t.\;y_{i}-[w^{T}\varphi (x_{i})+b]\leq \xi_{i},i=1,2,\ldots ,l \end{array},$$

where $\xi_{i}$ is the training error of vector $x_{i}$, φ(•) is the mapping from original feature space to reproducing kernel Hilbert space, C>0 is the regularization parameter. The dual problem of Equation (1) is:

(2) $$\begin{array}{l} \underset{\alpha }{\min}\;W_{SVC}=\frac{1}{2}\sum_{i=1}^{l}\sum_{j=1}^{l}\alpha_{i}Q_{i,j}\alpha_{j}-\;\sum_{i=1}^{l}\alpha_{i}+b\sum_{i=1}^{l}y_{i}\alpha_{i}\\ s.t.\;\sum_{i=1}^{l}y_{i}\alpha_{i}=0,\;0\leq \alpha_{i}\leq C,\;i=1,2,\ldots ,l \end{array},$$

where $Q_{i,j}=y_{i}y_{j}k\left(x_{i},x_{j}\right)$, $k\left(x_{i},x_{j}\right)$ is the kernel function satisfying the Mercer Theorem [29]. A commonly used kernel function is RBF kernel [43], which is formulated as:

(3) $$k(x_{i},x_{j})=\exp(-\gamma {\left\|x_{i}-x_{j}\right\|}^{2}).$$

After optimizing *W* with *α* and *b*, the indicator function of SVC can be rewritten as $f(x)=\mathrm{sgn}\left(\sum_{i=1}^{l}\alpha_{i}y_{i}k(x_{i},x)+b\right)$. During the optimization process of *W*, samples lying far away from the class boundary will be pruned by Karush-Kuhn-Tucker (KKT) conditions and their corresponding coefficients will be turned into zero [29]. Specifically, given a training sample $\left(x_{k},y_{k}\right)$, following condition function holds:

(4) $$y_{k}f(x_{k})-1\left\{ \begin{array}{l} \geq 0;\;\alpha_{k}=0\\ =0;\;0<\alpha_{k}<C\\ \leq 0;\;\alpha_{k}=C \end{array}\right..$$

Those samples with non-zero coefficients are named as support vectors (SVs). The dataset of support vectors (noted as *SV*) is a subset of original training dataset, and thus the predictive model of standard C-SVC is sparse, and the indicator function can be simplified as $f(x)=\mathrm{sgn}\left(\sum_{x_{i}\in SV}\alpha_{i}y_{i}k(x_{i},x)+b\right)$.

The standard C-SVC can not change its model when concept drift happens and thus shows nonadapting property and marks the performance degradation with time. To avoid ambiguity, we note the standard C-SVC as nonadapting SVC (NSVC) in this paper.

#### 2.2.2. Incremental SVM

**Figure 1:**
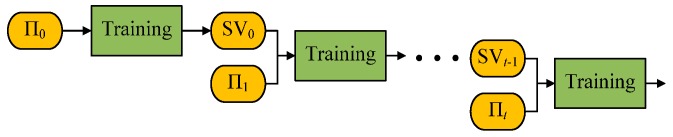
Generic schema of Incremental SVC. Support vectors (SVs) from previous predictive model are combined with newly coming samples to train a new predictive model and select new support vectors based on KKT conditions.

To change SVM into an adaptive classifier, a typical strategy is to update the predictive model with new samples, namely, learning incrementally. When the data come in the form of stream, they are segmented into different batches $\left\{\Pi_{1},\Pi_{2},\ldots \right\}$. Newly labeled data batch Π*_t_* and old support vector set SV*_t_*_−1_ will compose a new training set to retrain the predictive model and get new support vector set SV*_t_*. Figure 1 shows the schema of typical Incremental SVC (ISVC) [33].

**Figure 2:**
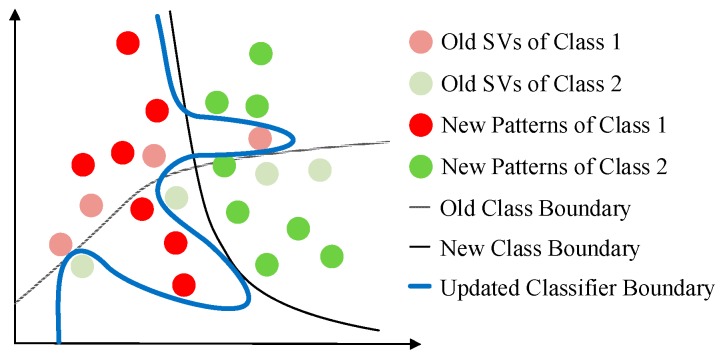
Schematic drawing to show the influence of KKT-conditions-based sample pruning on adaption. Old support vectors, which are close to the old class boundary, are more likely to be outdated according to the new class boundary, and to be added into the new predictive model, and thus to increase misclassification risk and complexity of the new predictive model.

Since the predictive model of ISVC is composed of support vectors (SVs), the penalizing mechanism for redundant information is based on KKT conditions [29]. However, KKT conditions can neither reduce the complexity of predictive model in long term, nor reduce the misclassification risk of new samples. Figure 2 shows the influence of KKT conditions on the adaption of ISVC. According to whether the boundary of classes changes or not, the concept drift can be categorized into *real concept drift* and *virtual drift* [19]. Only real concept drift has any influence on performance degradation. After real concept drift between two adjacent concepts, samples lying close to the old class boundary are more likely to be outdated according to the position of the new class boundary. Unfortunately, these samples would be kept as support vectors after penalizing of KKT conditions. Those outdated samples would increase the complexity and the misclassification risk of the predictive models in both supervised and unsupervised adaptive learning scenarios.

#### 2.2.3. Program Codes and Parameters of NSVC and ISVC

The codes of ISVC and NSVC were modified from libSVM [44]. To realize multi-class classifying, 1-against-1 strategy [45] was employed to get higher classification accuracy. Parameters *C* (relaxation parameter of SVM) and *γ* (RBF kernel parameter) were determined by *n*-fold cross validation [5] during the training process.

### 2.3. The Parcticle Adaptive Classifier

#### 2.3.1. General Structure of PAC Classifier

**Figure 3:**
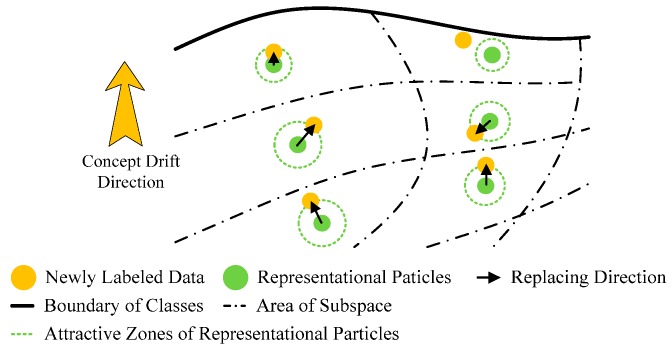
Schematic drawing to show the updating mechanism of PAC. Different from ISVC, the PAC strategy updates the predictive model by replacing old representative particles (RPs), other than adding new representative particles, to maintain the complexity of the predictive model. On the other hand, new samples close enough to one RP will replace the RP. The area lying in which the sample could replace the RP is the attractive zone of the RP. In this way, we could choose samples with high confidence to reduce the retraining risk.

According to above descriptions, ISVC has two shortcomings for concept adaption: (1) the rapid accumulation of the misclassification risk, and (2) the increasing complexity of the predictive model. In order to amend the shortcomings of ISVC, we proposed a novel adaptive learning structure for SVM, the particle adaption classifier (PAC). Figure 3 illustrates the working principles of PAC. The core concept of PAC is its replaceable support vectors, named as representative particles (RPs). Compared with SVs of NSVC or ISVC, RPs of PAC have the following characteristics:

(1) The distribution of RPs is sparse. Different from traditional KKT-conditions-based pruning, the sparsity of RPs is originated from the down sampling of original training dataset. The down sampling process will keep the distribution characteristics of the whole feature space, rather than the distribution characteristics of the class boundary. Specifically, each RP can be regarded as a center point of a subspace in the feature space, and thus the distribution density of RPs largely approximates the probability density function (PDF) of the feature space. The predictive model trained by RPs will have the similar classification performance with the predictive model trained by original training dataset, but have higher computational efficiency.
(2) With numerous replacements of RPs, the general predictive model is able to track the concept drift. Since the distribution density of RPs approximates the PDF of the feature space, the replacement for an RP with an appropriate new sample, is equivalent to update the PDF estimation of a subspace of the feature space. In this way, the classifier is able to track the concept drift.
(3) Based on RPs, it is possible to evaluate the misclassification risk of a new sample. When we try to evaluate the misclassification risk of a new sample in unsupervised adaptive learning scenarios, we have to establish some basic assumptions. Considering the property of EMG signals [5], at least two widely used assumptions on the class boundary are valid for the classification problem of EMG signals: (a) cluster assumption [39], and (b) smoothness assumption [39]. The cluster assumption assumes that two samples near enough in the feature space are likely to share the same label. The smoothness assumption assumes that the class boundary is smooth, namely, the sample far away from the class boundary is more likely to maintain the label after concept drifting, than samples near the class boundary. If a sample is close enough to an RP, and has the same predicted label with the RP, it is likely to be rightly classified. When pruning newly coming samples with their distances to RPs, it is possible to suppress the accumulation of misclassification risk. The area in which the new sample is adopted to replace old RP is the attractive zone of the RP.

**Figure 4:**
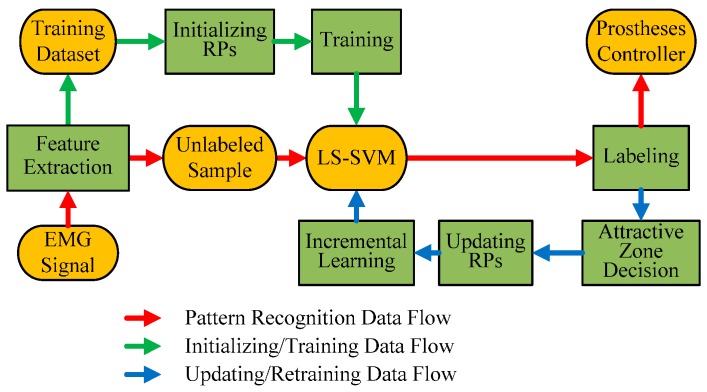
The learning structure of PAC. Different from ordinary pattern recognition method, PAC chooses RPs before training LS-SVM during the initialing process, and adds an updating process after labeling unlabeled samples. The updating process is composed of attractive zone decision (to select unlabeled samples with high confidence), updating RPs with selected samples, and universal incremental LS-SVM with updated RPs.

The size of the attractive zone is crucial, because it determines the tracking speed of PAC, the reliability of unlabeled new samples, and the distribution of the new group of RPs. Larger attractive zone will be able to track the concept drift with lager drift rate, but will also result in higher updating risk and worse representability of RPs. Furthermore, old RPs should have larger attractive zone than new RPs, because they are more likely to be outdated. Besides, the initialization of RPs and the choice of the predictive model are also essential for PAC. The initialization of RPs affects the representability of RPs, which will be discussed in details in Section 2.3.3. Since the RPs is sparse, it is better to choose a full-dense predictive model to maintain the sparsity as well as the representability of RPs. The full-dense property means that there is no further pruning mechanism when training the predictive model. In the case of PAC, the full-dense feature of predictive model refers that all RPs act as SVs for the predictive model. We chose LS-SVM as the full-dense predictive model [46] in this paper. To retrain the LS-SVM efficiently, we proposed the universal incremental LS-SVM for the first time, which could operate inserting, deleting and replacing support vectors in the predictive model conveniently. Figure 4 shows the general learning structure of proposed PAC.

#### 2.3.2. Universal Incremental LS-SVM

To solve the binary classification problem with a set of training pairs ${\left\{\left(x_{i},y_{i}\right)\right\}}_{i=1}^{l}$, $x_{i}\in R^{n}$, $y_{i}\in \{-1,+1\}$, LS-SVM [34] formulates the optimization primal problem as follows:

(5) $$\begin{array}{l} \underset{w,b,\xi }{\min}\;J_{LS}=\underset{w,b,\xi }{\min}\;(\frac{1}{2}w^{T}w+\frac{C}{2}\sum_{i=1}^{l}\xi_{i}^{2})\\ s.t.\;y_{i}-[w^{T}\varphi (x_{i})+b]=\xi_{i},i=1,2,\ldots ,N \end{array}.$$

where $\xi_{i}$ is the training error of vector $x_{i}$, φ(•) is the mapping from original feature space to reproducing kernel Hilbert space, C>0 is the regularization parameter. The Lagrangian for above optimization problem is:

(6) $$L(w,b,\xi )=J_{LS}-\sum_{i=1}^{l}\alpha_{i}\{[w^{T}\varphi (x_{i})+b]+\xi_{i}-y_{i}\}.$$

where $\alpha_{i}$ is the Lagrange multiplier of $x_{i}$. Conditions for the optimality of L(w,b,ξ) are:

(7) $$\left\{ \begin{array}{l} \partial L(w,b,\xi )/\partial w=0\to w=\sum_{i=1}^{l}\alpha_{i}\varphi (x_{i})\\ \partial L(w,b,\xi )/\partial b=0\to \sum_{i=1}^{l}\alpha_{i}=0\\ \partial L(w,b,\xi )/\partial \xi_{i}=0\to C\xi_{i}=\alpha_{i}\\ \partial L(w,b,\xi )/\partial \alpha_{i}=0\to \alpha_{i}[\sum_{j=1}^{l}\alpha_{j}k(x_{i},x_{j})+1/C]+b=y_{i} \end{array}\right..$$

where $k(x_{i},x_{j})=\varphi {\left(x_{i}\right)}^{T}\varphi (x_{j})$ is the kernel function similar to standard C-SVC. Equation (7) can be rewritten in the form of linear equations:

(8) $$\left[\begin{array}{cc} 0 & E_{V}{}^{T}\\ E_{V} & K+C^{-1}I \end{array}\right]\left[\begin{array}{c} b\\ \alpha \end{array}\right]=\left[\begin{array}{c} 0\\ Y \end{array}\right].$$

where $E_{V}={\left[1,1,\ldots ,1\right]}^{T}$, $\alpha ={\left[\alpha_{1},\alpha_{2},\ldots ,\alpha_{l}\right]}^{T}$, $Y={\left[y_{1},y_{2},\ldots ,y_{l}\right]}^{T}$, $\in R^{l\;\times \;1}$, ***I*** is an *l*-rank identity matrix, ***K*** is an *l*-rank kernel matrix with entries $K_{i,j}=\;k(x_{i},x_{j})$.

Let $H=K+C^{-1}I$, the solution of Equation (8) is:

(9) $$\{\begin{array}{c} b=\frac{E_{V}{}^{T}H^{-1}Y}{E_{V}{}^{T}H^{-1}E_{V}}\\ \alpha =H^{-1}(Y-bE_{V}) \end{array}.$$

The dual model of the indicator function can be written as $f(x)=\mathrm{sgn}\{[\sum_{i=1}^{l}\alpha_{i}k(x_{i},x)]+b\}$. The computational complexity of the linear equations indicated in Equation (9) is of order *O*(*l*^3^) [47]. The major complexity lies in the computing of ***H***^−1^, the inverse of an *l*-rank symmetric positive definite matrix [47].

With universal incremental learning method, the computational complexity for retraining the predictive model will be reduced to *O*(*l*^2^). The universal incremental LS-SVM is able to insert, delete and replace a sample in any position in the RP dataset. Before detailed description, we formulate the operation of inserting, deleting and replacing respectively. Given a RP dataset *S*(*l*) with *l* samples, and the *p*th sample ***x**_p_* (1 ≤ *p* ≤ *l*) of *S*(*l*), we define the dataset deleting ***x**_p_* from *S*(*l*) as *S*(*l* − 1). The operations and their corresponding learning process are defined as:

- Inserting: adding ***x**_p_* at the *p*th position into *S*(*l* − 1), is equivalent to training from *S*(*l* − 1) to *S*(*l*);
- Deleting: deleting ***x**_p_* at the *p*th position from *S*(*l*), is equivalent to training from *S*(*l*) to *S*(*l* − 1);
- Replacing: deleting ***x**_p_* at the *p*th position from *S*(*l*), adding **x**_p_* at the *p*th position into *S*(*l* − 1), is equivalent to the combination of training from *S*(*l*) to *S*(*l* − 1) and training from *S*(*l* − 1) to S*(*l*).

Since the training process from *S*(*l* − 1) to S*(*l*) is similar with the process from *S*(*l* − 1) to *S*(*l*), we only need to work out an algorithm to train from *S*(*l*) to *S*(*l* − 1) and from *S*(*l* − 1) to *S*(*l*). We note the ***H*** matrices for training classifiers with *S*(*l*) and *S*(*l* − 1) as ***H***(*l*) and ***H***(*l* − 1) respectively. ***H***(*l*) and ***H***(*l* − 1) can be divided as:

(10) $$H(l-1)=\left[\begin{array}{cc} H_{1} & H_{2}\\ H_{2}^{T} & H_{3} \end{array}\right],H(l)=\left[\begin{array}{ccc} H_{1} & h_{1} & H_{2}\\ h_{1}^{T} & h_{pp} & h_{2}^{T}\\ H_{2}^{T} & h_{2} & H_{3} \end{array}\right].$$

where $H_{1}\in R^{\left(p\;-\;1)\;\times \;(p\;-\;1\right)}$, $H_{2}\in R^{\left(p\;-\;1)\;\times \;(l\;-\;p\right)}$, $H_{3}\in R^{\left(l\;-\;p)\;\times \;(l\;-\;p\right)}$ are divisions of ***H***(*l* − 1) at *p*th row and *p*th column, ***h***_1_ = [*k*(***x***_1_, ***x**_p_*),…, *k*(***x**_p_*_− 1_, ***x**_p_*)]^T^, ***h***_2_ = [*k*(***x**_p_*_+ 1_, ***x**_p_*),…,*k*(***x***_l_, ***x**_p_*)]^T^, *h_pp_* = *k*(***x**_p_*, ***x**_p_*) + 1/*C*. According to the property of Cholesky factorization [48], ***H***(*l*) and ***H***(*l* − 1) are symmetric definite and hence they can be Cholesky factorized uniquely. We note the upper triangular matrices after Cholesky factorization as ***U*** and ***W***, and then we get $H(l-1)=W^{T}W,H(l)=U^{T}U$.

Similar to ***H***(*l*) and ***H***(*l* − 1), ***U*** and ***W*** can be written as:

(11) $$W=\left[\begin{array}{cc} W_{1} & W_{2}\\ O & W_{3} \end{array}\right],U=\left[\begin{array}{ccc} U_{1} & u_{1} & U_{2}\\ 0^{T} & u_{pp} & u_{2}^{T}\\ O & 0 & U_{3} \end{array}\right].$$

where $O\in R^{\left(p\;-\;1)\;\times \;(p\;-\;1\right)}$ is a matrix of zeros, ***0*** $\in R^{(p\;-\;1)\;\times \;1}$ is a vector of zeros. Because of the uniqueness of Cholesky factorization, we can compute from ***W*** to ***U*** as:

(12) $$\begin{array}{l} U_{1}=W_{1},\;U_{2}=W_{2},u_{1}={\left(U_{1}^{T}\right)}^{-1}h_{1}\\ u_{pp}=\sqrt{h_{pp}-u_{1}^{T}u_{1}}\\ u_{2}=(h_{2}-U_{2}^{T}u_{1})/u_{pp}\\ U_{3}^{T}U_{3}=W_{3}^{T}W_{3}-u_{2}u_{2}^{T} \end{array}.$$

On the other hand, we can compute from ***U*** to ***W*** as:

(13) $$\begin{array}{l} W_{1}=U_{1},\;W_{2}=\;U_{2}\\ W_{3}^{T}W_{3}=U_{3}^{T}U_{3}+\;u_{2}u_{2}^{T} \end{array}.$$

The detailed algorithms of the inserting process and the deleting process are show in Algorithm 1. In the algorithm, we retrain the predictive model without computing the inverses of large matrices, but with forward substitutions of triangular matrices and low-rank Cholesky downdating/updating of symmetric positive definite matrices. The computational complexity of forward substitution is of order *O*(*l*^2^), while the computational complexity of Cholesky updating/downdating is of order *O*[(*l* − *p*)^2^]. The replacing process is realized by one-step deleting and one-step inserting. Therefore, the overall computational complexity of universal incremental LS-SVM is of order *O*(*l*^2^), much lower than that of standard LS-SVM, which is of order *O*(*l*^3^).
**Algorithm 1.**
Algorithm for inserting and deleting processes of universal incremental LS-SVM.**Inserting****Deleting****Input**: $S(l-1)={\left\{\left(x_{i},y_{i}\right)\right\}}_{i=1,i\neq p}^{l}$, R(l−1), $\left(x_{p},y_{p}\right)$, 1≤p≤l, *C***Input**: $S(l)={\left\{\left(x_{i},y_{i}\right)\right\}}_{i=1}^{l}$, R(l), 1≤p≤l**Output:**
R(l), α(l), b(l)**Output:**
R(l−1), α(l−1), b(l−1)1W←R(l−1)1U←R(l)2$\left[\begin{array}{cc} W_{1} & W_{2}\\ O & W_{3} \end{array}\right]\leftarrow W$//divide at
*p*th row and clomn2$\left[\begin{array}{ccc} U_{1} & u_{1} & U_{2}\\ 0^{T} & u_{pp} & u_{2}^{T}\\ O & 0 & U_{3} \end{array}\right]\leftarrow U$//divide at
*p*th, *p* + 1st row and clomn3$h_{1}\leftarrow {\left[k\left(x_{1},x_{p}\right),\ldots ,k\left(x_{p-1},x_{p}\right)\right]}^{T}$3$W_{1}\leftarrow U_{1},\;W_{2}\leftarrow U_{2}$4$h_{2}\leftarrow {\left[k\left(x_{p+1},x_{p}\right),\ldots ,k\left(x_{l},x_{p}\right)\right]}^{T}$4$W_{3}^{T}W_{3}\leftarrow U_{3}^{T}U_{3}+u_{2}u_{2}^{T}$ //low-rank update5$h_{pp}\leftarrow k\left(x_{p},x_{p}\right)+1/C$5$W\leftarrow \left[\begin{array}{cc} W_{1} & W_{2}\\ O & W_{3} \end{array}\right]$6$U_{1}\leftarrow W_{1},\;U_{2}\leftarrow W_{2}$6$b(l-1)\leftarrow \frac{E_{V}{}^{T}W^{-1}{\left(W^{T}\right)}^{-1}Y}{E_{V}{}^{T}W^{-1}{\left(W^{T}\right)}^{-1}E_{V}}$7$u_{1}\leftarrow {\left(U_{1}^{T}\right)}^{-1}h_{1}$ //forward substitution7$\alpha (l-1)\leftarrow W^{-1}{\left(W^{T}\right)}^{-1}(Y-bE_{V})$8$u_{2}\leftarrow (h_{2}-U_{2}^{T}u_{1})/u_{pp}$8R(l−1)←W9$U_{3}^{T}U_{3}\leftarrow W_{3}^{T}W_{3}-u_{2}u_{2}^{T}$ //low-rank downdate10$U\leftarrow \left[\begin{array}{ccc} U_{1} & u_{1} & U_{2}\\ 0^{T} & u_{pp} & u_{2}^{T}\\ O & 0 & U_{3} \end{array}\right]$11$b(l)\leftarrow \frac{E_{V}{}^{T}U^{-1}{\left(U^{T}\right)}^{-1}Y}{E_{V}{}^{T}U^{-1}{\left(U^{T}\right)}^{-1}E_{V}}$//forward substitution12$\alpha (l)\leftarrow U^{-1}{\left(U^{T}\right)}^{-1}(Y-bE_{V})$//forward substitution13R(l)←U



#### 2.3.3. Initialization and Updating of RPs

The initialization of RPs affects the representability as well as the sparsity of RPs. Though many methods could initialize RPs appropriately, this paper initializes RPs with following two steps:

(a) Dividing the training dataset into *m* clusters based on kernel space distance and *k*-medoids clustering method [49];
(b) Randomly and proportionally picking samples from each cluster as RPs with a percentage *p*.

Here, choosing kernel space distance to cluster samples rather than the euclidean metric, is to reduce the time cost of computing attractive zone as well as to be consistent with the kernel trick in the predictive model. The kernel space distance between two vectors ***x**_i_* and ***x**_j_* is defined as:

(14) $$d(x_{i},x_{j})=\sqrt{{\left\|\phi (x_{i})-\phi (x_{j})\right\|}^{2}}=\sqrt{k(x_{i},x_{i})+k(x_{j},x_{j})-2k(x_{i},x_{j})}.$$

If we choose RBF kernel, namely, $k(x_{i},x_{j})=\exp(-\gamma {\left\|x_{i}-x_{j}\right\|}^{2})$, where *γ* is the kernel parameter, the kernel space distance can be simplified as:

(15) $$d(x_{i},x_{j})=\sqrt{2-2k(x_{i},x_{j})}.$$

When determine the attractive zone of RPs, following principles are taken into consideration:

(a) The new sample is close enough to its nearest RP;
(b) Older RP is more likely to be replaced;
(c) The new sample is in the same class with its nearest RP (for supervised adaption only).

Therefore, for the new sample ***x**_N_*, we firstly find out the nearest RP to ***x**_N_* according to the kernel space distance and the timing coefficient, as:

(16) $$I=\underset{i}{\arg}\left\{\underset{i}{\min}[\exp(t_{i}/\lambda )d(x_{N},x_{i})]\right\}.$$

where *I* is the index of the nearest RP to ***x**_N_*, ***x**_i_* is the *i*th RP, *t_i_* is the unchanging time from last time ***x**_i_* being replaced, *λ* is the factor controlling the influence of time. Then we use the threshold *d_Th_* to determine whether ***x**_N_* is close enough to its nearest RP, ***x**_I_*, or not:

(17) $$D(x_{N},x_{I})=d_{Th}-\exp(t_{I}/\lambda )d(x_{N},x_{I})\left\{ \begin{array}{ll} >0 & \mathrm{to}\;\mathrm{replace}\;x_{I}\;\mathrm{with}\;x_{N},\;t_{I}=0\\ \leq 0 & \mathrm{to}\;\mathrm{ignore}\;x_{N},\;\mathrm{all}\;t_{i}=t_{i}+1 \end{array}\right..$$

Considering that kernel product $k(x_{N},x_{i})$ is computed for making the prediction, not much extra computational burden is added for the decision function. The replacing of the representational particle is computed with the algorithm of universal incremental LS-SVM. The detailed algorithm for uPAC is shown in Algorithm 2.
**Algorithm 2.** Initializing and updating algorithm for uPAC.**Require:**
*m* > 0, *p* > 0, *d_Th_* > 0, λ > 01**Cluster** training dataset into *m* clusters //*k*-medoids clustering2**Extract** representative particles with percentage *p* into dataset *RP*3**Train** LS-SVM predictive model *PM* with *RP*4**while *x****_N_* is valid **do**5 $y_{N}\leftarrow f(PM,x_{N})$ //predict label6 **all***t_i_*←*t_i_* + 1 //update unchanging time7 $I\leftarrow \underset{i}{\arg}\left\{\underset{i}{\min}[\exp(t_{i}/\lambda )d(x_{N},x_{i})]\right\}$8 $D\leftarrow d_{Th}-\exp(t_{I}/\lambda )d(x_{N},x_{I})$9 **if**
*D* > 010  *t_I_*←0 //clear unchanging time11  ***x**_I_* ←***x**_N_* //update RP12  **Update**
*PM* //replcaing with universal incremental LS-SVM13 **end if**14**end while**

#### 2.3.4. Program Codes and Parameters of PAC

**Figure 5:**
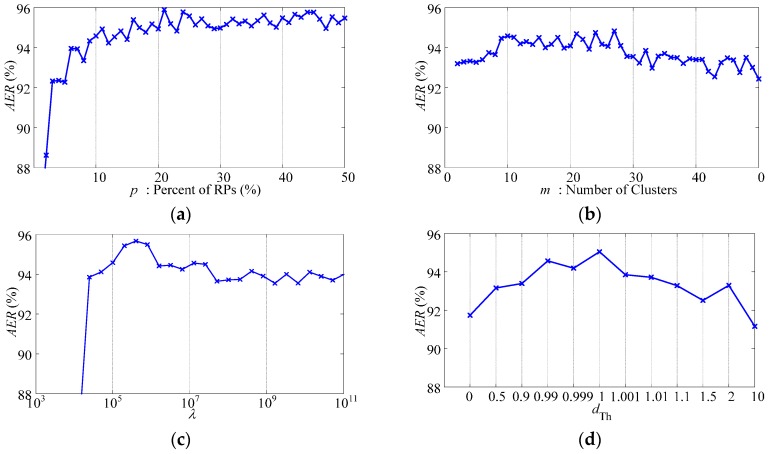
Classification performance with varying classifier parameters. (**a**) Varying *p* with *m* = 10, *λ* = 10^5^, and *d_Th_* = 0.99; (**b**) Varying *m* with *p* = 10%, *λ* = 10^5^, and *d_Th_* = 0.99; (**c**) Varying *λ* with *p* = 10%, *m* = 10, and *d_Th_* = 0.99; (**d**) Varying *d_Th_* with *p* = 10%, *m* = 10, and *λ* = 10^5^.

To realize multi-class classifying, one-against-one strategy is employed to get higher classification accuracy [45]. Parameters *C* (relaxation parameter of LS-SVM) and *γ* (RBF kernel parameter), which control the generalization of LS-SVM, are determined by *n*-fold cross validation during the training process of LS-SVM. Parameters *m* and *p* control the sparsity and the representability of RPs. Parameters *d_Th_* and *λ* balance the ability to track concept drifts with large drift rate and the ability to choose samples with high confidence. When determining the parameters *m*, *p*, *d_Th_*, and *λ*, we use one trial of data described in Section 2.5.4, based on the measure of *AER* described in Section 3.3.1. Figure 5 illustrates the classification performance with varying classifier parameters. For different parameters, the performance shows different changing tendencies as follows:

- Parameter *p*: With the increasing of *p*, the ending accuracy *AER* of the data sequence increases, while the slope of the increasing decreases. Such changing tendency indicates that PAC with redundant RPs is not only inefficient but also unnecessary. The recommended interval for *p* is between 10% and 20%.
- Parameter *m*: The adjustment of *m* changes the performance slightly. With the increasing of *m*, the performance experiences slight improvement at first and then slight deterioration. It has an optimal interval as between 9 and 28.
- Parameter *λ*: The choices of *d_Th_* and *λ* are highly related. There is an optimal interval for *λ* as between 10^4^ and 10^6^, when *d_Th_* is chosen as 0.99. On one hand, the choices of *λ* below the lower boundary of the optimal interval will result in indiscriminate replacement of RPs and complete failure of the classifier. On the other hand, the choices of *λ* higher than the upper boundary of the optimal interval will weaken the influence of time and result in slight deterioration.
- Parameter *d_Th_*: Similar to *λ*, there is an optimal interval for *d_Th_* as between 0.9 and 1.1, when *λ* is chosen as 10^5^. It is remarkable that, because we choose the nearest RP of the new sample to be replaced, the classifier does not complete fail even when *d_Th_* is set as 0.

As shown in Figure 5, impropriate *p* and *λ* will result in complete failure of the classifier. Therefore, when choosing the classifier parameters, we should choose *p* and *λ* firstly, and then choose the other two parameters accordingly. However, it remains an open problem to avoid over fitting and local optimum during the optimizing process of the parameters. Since this paper is to prove the potentiality of PAC classifier, after trying some groups of PAC parameters, we choose one group of PAC parameters with moderate performance, and compare the performance with ISVC and NSVC. The chosen group of PAC parameters are as, *m* = 10, *p* = 10%, *d_Th_* = 0.99, and *λ* = 10^5^.

**Figure 6:**
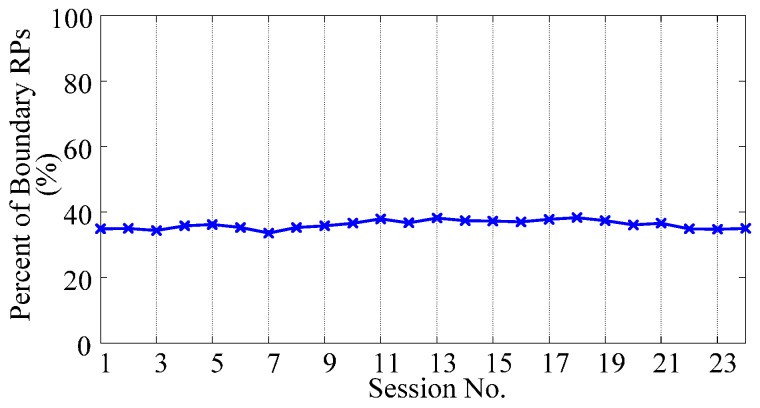
Proportion of boundary RPs to all RPs during the adaptive learning process.

Furthermore, to illustrate the distribution of RPs, we measure the proportion of boundary RPs to all RPs, by training the NSVC with RPs. After training, RPs with non-zero coefficients are regarded as boundary RPs. Figure 6 shows the changing tendency of the proportion of boundary RPs. Session 1 is the initializing session for PAC; Sessions 2 to 24 are the validating sessions. As shown in the figure, the proportion of boundary RPs maintains less than 40% of all RPs, which satisfies the requirement of the respresentability.

### 2.4. Performance Validation for Adaptive Learning

#### 2.4.1. Supervised and Unsupervised Adaptive Learning Scenarios

Label information is overwhelmingly important for adaptive learning [19]. For an adaptive learner, adapting processes with complete, timely supervised label information represents the *ideal case* in which the learner can achieve its best performance, whereas adapting processes with none of supervised label information represents the *realistic case* in which the learner shows its basic adaptability. Above two kinds of adapting processes are defined as supervised adaptive learning scenarios and unsupervised adaptive learning scenarios respectively.

PAC working in unsupervised adaptive learning scenarios is the original algorithm shown in Algorithm 2, whereas PAC working in supervised adaptive learning scenarios introduces a pruning strategy that ignores misclassified samples when choosing and updating RPs to maintain high confidence of the updating. ISVC working in unsupervised adaptive learning scenarios is to retrain predictive model with output predictions of the old predictive model, whereas ISVC working in supervised adaptive learning scenarios is to retrain predictive model with supervised labels. PAC working in supervised and unsupervised adaptive learning scenarios are named as sAPC and uPAC respectively, whereas ISVC working in supervised and unsupervised adaptive learning scenarios are named as sISVC and uISVC respectively.

#### 2.4.2. Data Organization for Validation

**Figure 7:**
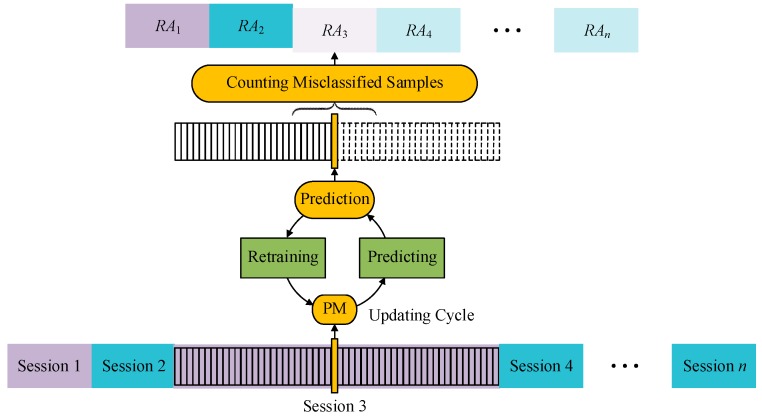
The verification process for adaptive learning classifiers. Samples with the same concept compose a session. Sessions with different concepts compose a session sequence. Samples in the session sequence are fed to the updating cycle one by one, and recognition accuracy (*RA_i_*) is concluded in the unit of session. During an updating cycle, we firstly use the predictive model (PM) to classify the sample and make the prediction, then use the sample and its corresponding supervised label information (in supervised adaptive learning scenarios) or prediction result (in unsupervised adaptive learning scenarios) to retrain the predictive model.

In this paper, we define a *session* as a set of data samples with the same concept. And thus a *session sequence* is defined as a series of sessions with varying concept. The minimum unit to measure the recognition accuracy and the concept drift is a session. It is essential to examine the variation of recognition accuracy in a session sequence. The actual verification process is illustrated in Figure 7. For a session sequence with *n* sessions, samples are aligned into a line with the session number and fed to the updating cycle one sample by one sample. The misclassified samples are counted in unit of sessions. The rate of correctly labeled samples to all samples in one session is defined as the value of recognition accuracy.

To thoroughly compare the performance of PAC with ISVC and NSVC, we conducted experiments based on both simulated data with controlled concept drift and continuously acquired realistic one-day-long data to explore both the theoretical difference in adaptability and the empirical difference in clinical use.

### 2.5. Experimental Setup

#### 2.5.1. EMG Data Recording and Processing

**Figure 8:**
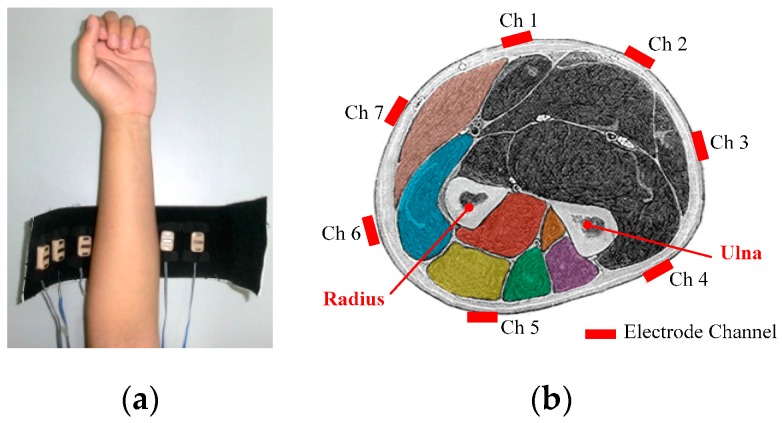
Layout of EMG electrodes. (**a**) Axial layout of EMG electrodes; (**b**) radial layout of EMG electrodes.

EMG signals were acquired by seven commercial electrodes (Otto Bock, Vienna, Austria [50]) which output regularized (0–5 V) and smoothed root mean square (RMS) features of EMG signals [6], rather than raw EMG signals. We employed the output of the electrodes as EMG signal features directly. Since the bandwidth of the output signal feature was in the range of 0–25 Hz [51], the features were acquired at the rate of 100 Hz, as researches in [6,51,52]. The electrodes were placed around the forearm, apex of the forearm muscle bulge, with a uniform distribution. The layout of the electrodes is shown in Figure 8.

**Figure 9:**
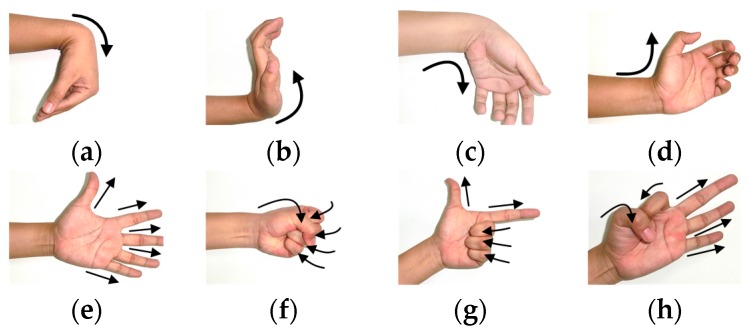
Motions during EMG data acquiring experiments. (**a**) Wrist flexion (WF); (**b**) wrist extension (WE); (**c**) ulnar deviation (UD); (**d**) radial deviation (RD); (**e**) all finger extension (AE); (**f**) all finger flexion (AF); (**g**) thumb-index extension (TE); (**h**) thumb-index flexion (TF). Motions (**a**–**d**) are four typical wrist movements, whereas motions (**e**–**h**) are four typical hand movements.

During the experiment, eight typical hand and wrist movements shown in Figure 9 were measured. The eight motions could be categorized into two classes: motions a–d were four typical wrist motions whereas motions e–h were four typical hand motions. It would prove the applicability of adaptive learners on the prosthetic hand-wrist system if the adaptive learners could discriminate the eight motions properly. Furthermore, the four hand motions could be regarded as two grasp/open pairs: powerful grasp and precise grasp [53], which are essential hand movements in activities of daily life (ADLs).

Since transient EMG signals contains more useful information than stable EMG signals [54], we trained and verified the classifier with acquired transient EMG signals. To eliminate the interference of weak contractions, only those contractions larger than 20% of the maximum voluntary contraction (MVC) were recorded. Specifically, before the principal experiment, we acquired 100 samples for each kind of motions while subjects conducted maximum voluntary contractions, and set an averaged value for one subject (*MVC_i_*, *i* corresponding to the serial number of the subject) over all samples, all motions and all channels. During principal experiment, the averaging value over all channels of a sample would be checked with its corresponding *MVC_i_* and would be recorded only if it was larger than 20% of *MVC_i_*. During recording process, a progress bar was shown to the subject to indicate how many valid samples had been recorded.

The data were acquired in units of sessions. Every session included 4000 samples: 500 samples for each of the eight motions. Acquiring the data of one session needed around 3 min. After acquisition, all the data were processed by PAC, ISVC and NSVC on a PC computer with the CPU of 3.2 GHz and memory of 8 GB.

#### 2.5.2. Subjects

Eight able-bodied subjects (five men and three women, all right-handed) participated in the experiments. Their average age was 24.25 ± 2.12 years old and their average body mass index (BMI, weight/height^2^) was 21.84 ± 2.99 kg/m^2^. All the experimental protocols of this study were approved by the Ethical Committee of Harbin Institute of Technology and conformed to the Declaration of Helsinki. All subjects were fully informed about the procedures, risk, and benefits of the study, and written informed consent was obtained from all subjects before the study.

#### 2.5.3. Experiment I: Simulated Data Validation

One important measure on the performance of an adaptive learning method is the adaptability, referring to the tracking ability of the algorithm when concept drifts of different rates and extents happen. Without the loss of generality, linear time varying shift functions, which could tune both the extent and the rate of the drift of the concept, were added to raw EMG signals, to simulate the possible concept drift of EMG signals. Dataset of one session was chosen as the original data, noted as $E_{\left(0\right)}={\text{\{}x_{(0)i}\text{\}}}_{i\;=\;1}^{4000}$, whereas session with maximum simulated concept drift extent was noted as $E_{\left(1\right)}={\text{\{}x_{(1)i}\text{\}}}_{i\;=\;1}^{4000}$. Therefore, a simulated session sequence, within which a following session was generated by adding a portion of maximum drift extent to its preceding session, is noted as $Q_{n}={\text{\{}E_{\left(k/n\right)}\text{\}}}_{k\;=\;0}^{n}$, where *n* is the total number of divisions of maximum drift extent, *k* is the serial number of the session in the session sequence. Within a session sequence, larger *k* corresponded to larger drift extent from session *E*_(0)_. Two sessions in different session sequences, sharing the same value of *k/n*, had the same drift extent from session *E*_(0)_.

Specifically, to simulate the actual concept drift of EMG signals, simulated shifts were composed of two steps: (a) decreasing the signal-noise ratio (SNR) of every channel of EMG sensors to simulate the muscular fatigure or the change of skin-electrode impedance; (b) fusing the signal of adjacent two channels of EMG sensors to simulate the minor shift of electrodes. For the data session $E_{\left(k/n\right)}$ in the session sequence *Q_n_*, signals of each channel were computed as:

(18) $$x_{(k/n)j}=\left\{ \begin{array}{ll} {\tilde{x}}_{(k/n)j}\left[1-0.5(k/n)\right]+0.5{\tilde{x}}_{(k/n)j+1}(k/n), & j\neq 7\\ {\tilde{x}}_{(k/n)j}\left[1-0.5(k/n)\right]+0.5{\tilde{x}}_{(k/n)1}(k/n), & j=7 \end{array}\right..$$

where ${\tilde{x}}_{(k/n)j}=\frac{x_{(0)j}+0.25(k/n)}{1+0.25(k/n)}$ was the signal with direct current noise. Therefore, for the data session with maximum concept drift, the signals were generated as:

(19) $$x_{(1)j}=\left\{ \begin{array}{ll} 0.4x_{(0)j}+0.4x_{(0)j+1}+0.2, & j\neq 7\\ 0.4x_{(0)j}+0.4x_{(0)1}+0.2, & j=7 \end{array}\right..$$

During experiment, session sequences with *n* = 16, 18, 20, 22, 27, 32, 64, 128 were generated. When verifying the adaptive classifiers with a session sequence, we used original session $E_{\left(0\right)}$ to initialize the classifiers, and used the whole session sequence to realize performance validation as described in Figure 7.

#### 2.5.4. Experiment II: One-Day-Long Data Validation

To verify the performance of adaptive learning methods in clinical use, eight subjects participated in one-day-long data acquiring experiment. Each subject completed one trial. Therefore, we got eight trials of one-day-long EMG data acquisitions in total. During one trial, subjects conducted the electrodes donning before 9:30 (24 h clock, hereinafter) and the doffing after 22:00, and no extra donning and doffing was conducted during one trial. After electrodes prepared, the data sessions were recorded every half an hour from 10:00 to 21:30. Hence, after a one-day-long data acquiring trial, we got a total of 24 sessions, naming them from Sessions 1 to 24. In the intervals of the recording, the subjects could move their arms freely without detaching the electrodes.

**Figure 10:**
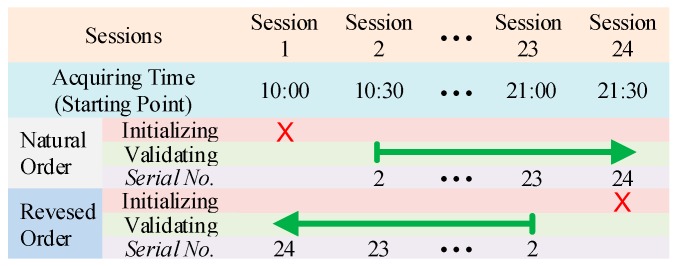
Validation method for acquired session sequence. For each sequence acquired by one-day-long experiment, sessions were organized in both the natural order (following the acquiring time) and the reversed order of the natural order.

Compared with the total acquiring time, the elapsing time within one session was ignorable. Therefore, the data in one session were regarded as data with the same concept. In this way the one-day-long data composed a session sequence. In order to reduce the influence of accidental factors on initializing session, as well as to maintain sequence length and maximum drift extent from the beginning of the sequence to the end of the sequence, we used two validating order for every session sequence. Figure 10 illustrated the validating order of an acquired data sequence.

In natural order, we used Session 1 to initialize classifiers, and Sessions 2 to 24 to compose the validating session sequence according to the acquiring time. In reversed order, session order in the sequence were reversed, namely, Session 24 acting as initializing session, Sessions 23 to 1 composing the validating session sequence. In order to avoid ambiguity, we endowed the recognition accuracy *RA* of a session in the validating sequence with a *serial number* as the corner mark, *RA_i_*. The serial number corresponded to the position of the session in the validating sequence. For example, the recognition accuracy of Session 2 in natural order was noted as *RA*_2_, whereas in reversed order it was noted as *RA*_23_.

## 3. Results

### 3.1. Support Vectors and Time Cost

**Figure 11:**
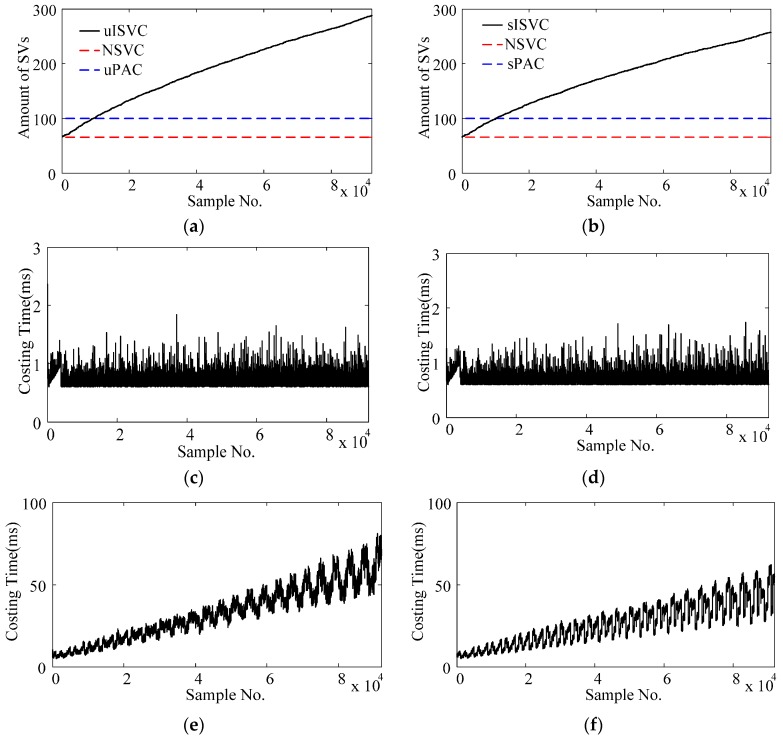
Amount of support vectors and time costs of updating cycles for adaptive classifiers. (**a**) Amounts of support vectors of uPAC, uISVC and NSVC; (**b**) Amounts of support vectors of sPAC, sISVC and NSVC; (**c**) Time cost per updating cycle of uPAC; (**d**) Time cost per updating cycle of sPAC; (**e**) Time cost per updating cycle of uISVC; (**f**) Time cost per updating cycle of sISVC. An updating cycle included predicting the sample label and retraining the predictive model. Time cost per updating cycle of sPAC and uPAC maintained less than 2 ms whereas that of sISVC and uISVC increased from 10 to 50 ms along with retraining times.

When validating the performance of adaptive classifiers, we recorded the amount of support vectors and the time cost per updating cycle along with the retraining times. Figure 11 shows the average variations during the classification tasks of one-day-long EMG data by averaging the eight subjects and both natural and reversed validating sequences. As shown in Figure 11a,b, both sISVC and uISVC increased their amounts of SVs, from less than the amounts of uPAC and sPAC to more than two times of the amounts uPAC and sPAC after 92,000 times of retraining. Furthermore, the increment of sISVC was less than that of uISVC. As the amount of SVs increased, the time cost per updating cycle of sISVC and uISVC increased accordingly, from 10 ms to more than 50 ms. At the same time, the time cost per updating cycle of uPAC and sPAC maintained less than 2 ms. Figure 11 illustrates that the proposed PAC has higher computational efficiency than ISVC and meets the requirement of online learning.

### 3.2. Classification Performance for Simulated Data

**Figure 12:**
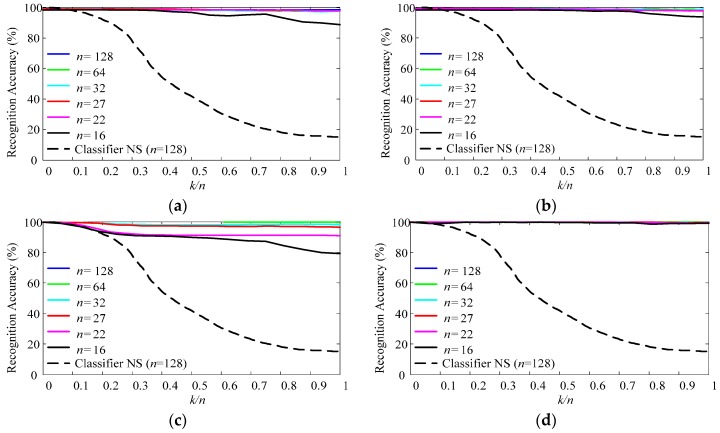
Recognition accuracy of simulated session sequences. (**a**) Recognition accuracy of uPAC (compared with NSVC); (**b**) Recognition accuracy of sPAC (compared with NSVC); (**c**) Recognition accuracy of uISVC (compared with NSVC); (**d**) Recognition accuracy of sISVC (compared with Classifier NSVC). Classifier NS is short for NSVC. Each line represents a session sequence. All the simulated session sequences share the same maximum drift extent (*DE*_max_), whereas amounts of sessions in the session sequences are different and noted as *n*. Since *DE*_max_ is fixed, for a session sequence, larger *n* corresponds to smaller drift rate of the session sequence. Parameter *k* is the serial number of a session in the session sequence. Within a session sequence, larger *k* corresponds to larger drift extent from session *E*_(0)_. If two sessions in different session sequences shares the same value of *k/n*, they have the same drift extent from session *E*_(0)_.

Figure 12a–d shows the validation results with simulated data. Within each figure, the solid lines illustrate the recognition accuracy of validating sequences classified by a specified adaptive classifier, whereas the dot line mark the recognition accuracy of the validating sequence with *n* = 128 classified by NSVC. From Figure 12a–d, the adaptive classifiers are uPAC, sPAC, uISVC, and sISVC respectively.

**Figure 13:**
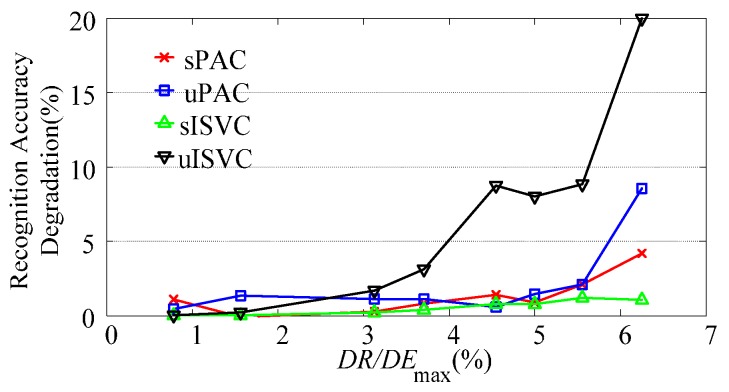
The recognition accuracy degradation of adaptive classifiers changing along with the ratio of drift rate (*DR*) to maximum drift extent (*DE*_max_). In this figure, each line represents the accuracy degradation of a type of adaptive classifier, and each point on the line represents the accuracy degradation of a session sequence. A session sequence is determined by the ratio of *DR* to *DE*_max_, which equals 1/*n*. Considering that all sessions share the same *DE*_max_, this figure shows the changing tendency of performance degradation along with drift rate. The recognition accuracy degradation is computed as the error rate of *E*_(1)_ in the session sequence.

As shown in Figure 12, when drift extent increased from 0 to its maximum extent, the recognition accuracy of NSVC decreased from nearly 100% for *E*_(0)_ to less than 20% for *E*_(1)_. The recognition accuracy of adaptive classifiers either maintained stable or decreased a little, keeping higher recognition accuracy than NSVC. To get more details about the accuracy variation with different drift rates, we focused on recognition accuracy degradation from *E*_(0)_ to *E*_(1)_, as shown in Figure 13.

As shown in Figure 13, the recognition accuracy degradation was related to the drift rate of a session sequence. Larger drift rate resulted in larger recognition accuracy degradation. Moreover, for one validating sequence, the recognition accuracy degradation of different adaptive classifiers increased with the order of sISVC, sPAC, uPAC, and uISVC.

### 3.3. Classification Performance for One-Day-Long Data

#### 3.3.1. Overall Performance

**Figure 14:**
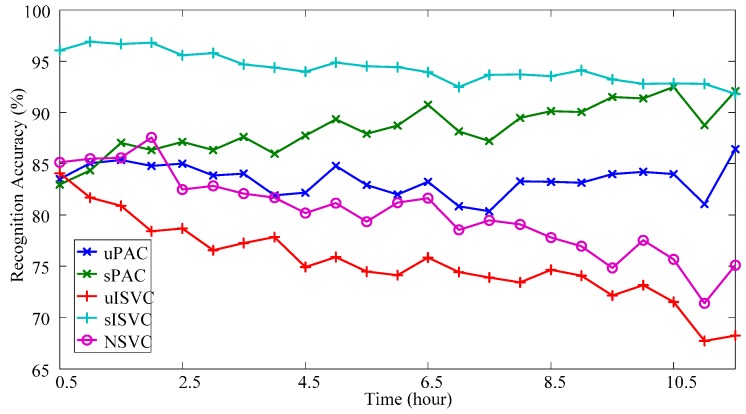
Recognition accuracy of realistic data from one-day-long EMG acquiring trials. The recognition accuracy in the validating session sequence are arranged according to the temporal distances from their corresponding sessions to the training session.

Figure 14 shows the accuracy variations for one-day-long data by averaging the classification results over all validating session sequences and all motion classes. As shown in the figure, at the beginning of the day, all classifiers with exception of sISVC shared the similar performance at around 85%. Specifically, the classification accuracy of the first session in a validating sequence was 83.48% ± 11.08% (Mean ± SD, similarly hereinafter) for uPAC, 82.94% ± 11.08% for sPAC, 84.04% ± 14.73% for uISVC, 85.12% ± 13.40% for NSVC, and 96.02% ± 5.30% for sISVC. As time went by, the difference between classifiers increased. By the end of the day, uPAC and sISVC almost maintained the performance, NSVC and uISVC experienced performance degradation to less than 75%, whereas sPAC experienced performance improvement to more than 90%.

To reduce the randomized error, we averaged the classification accuracy of last five sessions to describe the performance of a classifier at the end of the day, which was noted as *AER* (Average Ending Recognition Accuracy). Given the recognition accuracy of *i*th session in a validating sequence, *RA_i_*, the value of *AER* of a validating sequence was computed as:

(20) $$AER=\frac{1}{5}\sum_{i=20}^{24}RA_{i}\times 100\%.$$

**Figure 15:**
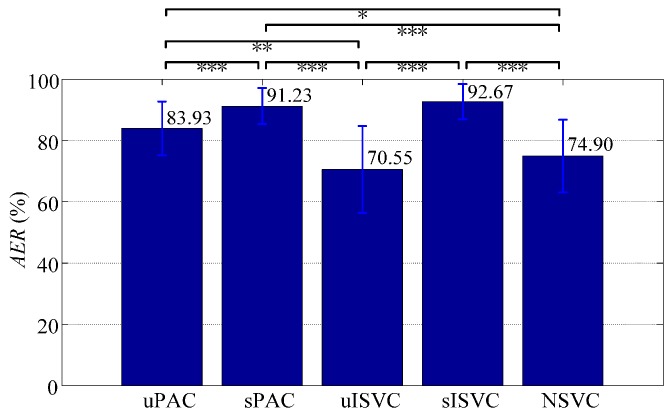
Statistics of all classifiers with the measure of *AER*. Each validating sequence was regarded as a repeated measure. Repeated measure ANOVA and post-hoc multiple comparisons were applied to examine the significance of differences. (***, *p* < 0.001; **, *p*<0.01; *, *p* < 0.05).

According to *AER*, the performances of classifiers at the end of the day were 83.93% ± 8.79% for uPAC, 91.23% ± 5.84% for sPAC, 70.55% ± 14.22% for uISVC, 92.67% ± 5.71% for sISVC, and 74.90% ± 11.84% for NSVC, respectively. A repeated measure ANOVA together with post hoc test with Bonferroni correction was employed to examine the overall and pairwise significance of all classifies in *AER*. Based on the repeated measure ANOVA test with Greenhouse-Geisser correction, the overall difference among all classifiers were significant (*p* < 0.001). Based on post-hoc multiple comparisons with Bonferroni correction of $\left(\begin{array}{l} 5\\ 2 \end{array}\right)=10$ comparing pairs, the significance of the pairwise differences is shown in Figure 15.

According to *AER*-based paired wise comparisons, we could reach following conclusions:

(1) Classifiers uPAC, sPAC and sISVC significantly improved the performance at the end of the day from NSVC by 9.03% ± 2.23% (*p* = 0.011, after Bonferroni correction of 10 comparison pairs, hereinafter), 16.32% ± 2.26% (*p* < 0.001), 17.77% ± 2.52% (*p* < 0.001) respectively, whereas uISVC showed no significant difference with NSVC (*p* = 0.857).
(2) Supervised adaptive learning scenarios had great superiorities on the performance over unsupervised adaptive learning scenarios. Specifically, sPAC had superiority over uPAC by 7.30% ± 1.10% (*p* < 0.001), and sISVC had superiority over uISVC by 22.12% ± 2.67% (*p* < 0.001). The conclusion is consistent with previous researches. But we can also found that the difference between sISVC and uISVC was much larger than the difference between sPAC and uPAC.
(3) In unsupervised adaptive learning scenarios, PAC was superior to ISVC, and in supervised adaptive learning scenarios, PAC was competitive with ISVC. Specifically, for sessions at the end of the day, the recognition accuracy of uPAC was higher than that of uISVC by 13.38% ± 2.62% (*p* = 0.001), whereas the recognition accuracy of sPAC had no significant difference with that of sISVC (*p* = 1).

#### 3.3.2. Performance Diversity of Different Motion Types

**Figure 16:**
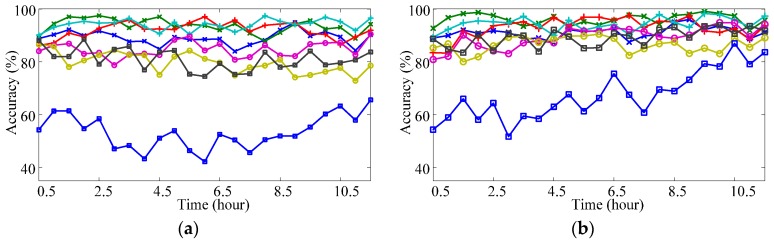

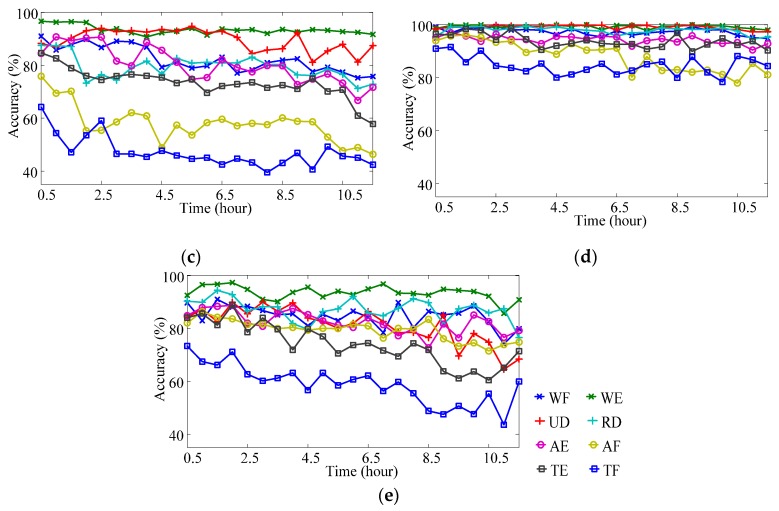
Accuracy variations for different motion types. (**a**) Classifier uPAC; (**b**) Classifier sPAC; (**c**) Classifier uISVC; (**d**) Classifier sISVC; (**e**) Classifier NSVC. The recognition accuracy is arranged according to the temporal distances from their corresponding sessions to the training session. (WF, wrist flexion; WE, wrist extension; UD, ulnar deviation; RD, radial deviation; AE, all finger extension; AF, all finger flexion; TE, thumb-index extension; TF, thumb-index flexion).

Figure 16 shows the accuracy variations of different motion types within one-day-long data by averaging the classifying results of all validating session sequences. As shown in the figure, motion TF (thumb-index flexion) had the worst classification results compared with other motion types. The classifier would have better overall performance if it improved the classification accuracy of motion TF. The classification of motion TF with different classifiers from the beginning of the day to end of day showed totally different changing trends: (1) NSVC and uISVC experienced decreasing more than 20%; (2) sISVC and uPAC almost maintained the accuracy with the variation less than 10%; (3) sPAC experienced increasing more than 20%.

**Figure 17:**
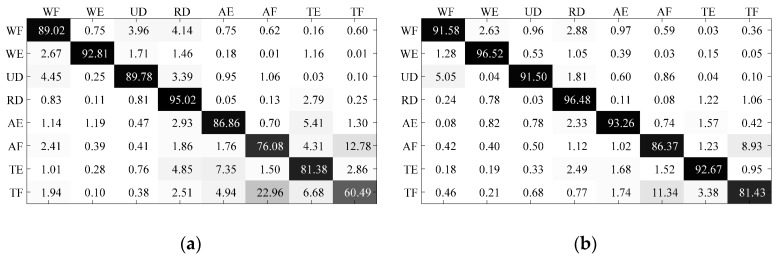

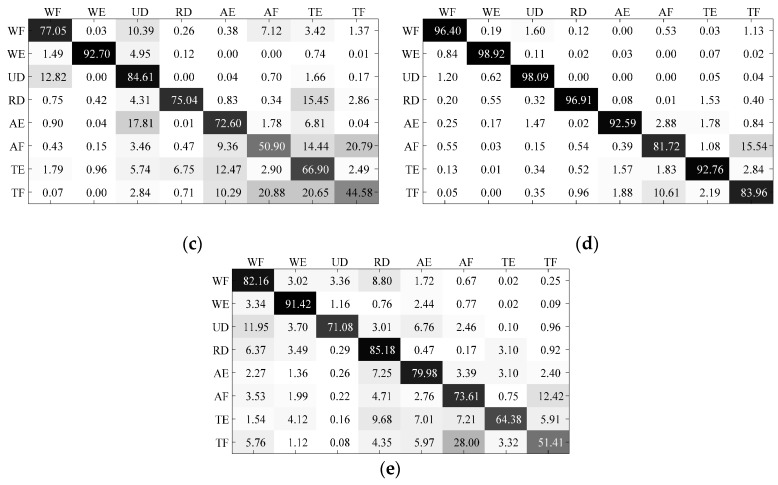
Confusion matrices for data of different motion types. (**a**) Classified by uPAC; (**b**) Classified by sPAC; (**c**) Classified by uISVC; (**d**) Classified by sISVC; (**e**) Classified by NSVC. The confusion matrices are computed by averaging the classification results of last five sessions in a validating sequence at first, and then averaging all validating sequences.

In order to analyze the source of misclassifications, Figure 17 illustrates the confusion matrices of motion classification with different classifiers. In the confusion matrix noted as ***CM***, each element ***CM***(*i*, *j*) represents the percentage of samples with ground truth class *i* being classified into class *j*, and thus the sum value of entries in one row always satisfies $\sum_{j}\mathrm{CM}(i,j)=100\%$. The confusion matrices shown in Figure 17 are computed by averaging the classification results of last five sessions in a validating sequence at first, and then averaging all validating sequences.

The non-diagonal elements in the confusion matrices could be seen as the components of misclassifications. As shown in the figure, the confusions between AF and TF were the largest components of misclassifications of all classifiers, which implied that classifying motions AF and TF was more difficult than other classifying problems. Considering the different classification results of different motions, it is necessary to take the motion diversity into consideration when comparing the performance of classifiers.

A safe way is to check the motions with the worst recognition accuracy. Therefore, we average the classification accuracy of motions with lowest recognition accuracy in last five sessions, noted as *WMP* (Worst Motion Performance). Given the recognition accuracy of *j*th class of *k*th session in a validating sequence as *RA_k_*_,*j*_, the value of *WMP* of the validating sequence is computed as:

(21) $$WMP=\frac{1}{5}\sum_{k=20}^{24}\left(\underset{j}{\min}RA_{k,j}\right)\;\times \;100\%$$

**Figure 18:**
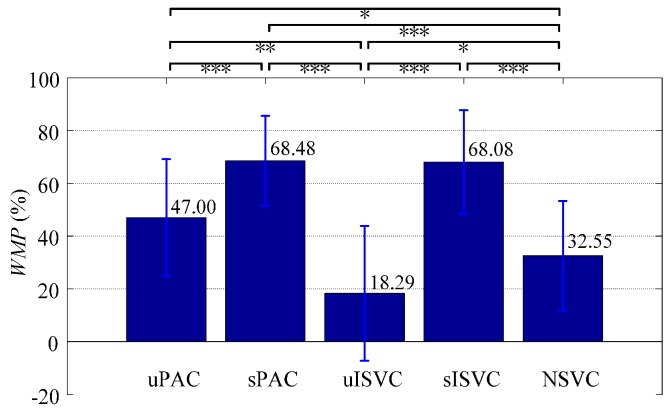
Statistics of *WMP* for all classifiers. Each validating sequence was regarded as a repeated measure. Repeated measure ANOVA and post-hoc multiple comparisons were applied to examine the significant differences after pairwise comparing. (***, *p* < 0.001; **, *p* < 0.01; *, *p* < 0.05).

According to the measure of *WMP*, the lowest motion recognition accuracy of the classifier was 47.00% ± 22.84% for uPAC, 68.48% ± 17.57% for sPAC, 18.29% ± 26.43% for uISVC, 68.08% ± 20.25% for sISVC, and 32.55% ± 21.48% for NSVC respectively. The *WMP*-based comparisons showed similar results with *AER*-based comparisons as shown in Figure 18, and the conclusions were as follows:

(1) Classifiers uPAC, sPAC and sISVC significantly improved *WMP* from NSVC by 14.45% ± 4.06% (*p* = 0.028), 35.93% ± 3.79% (*p* < 0.001), 35.53% ± 4.94% (*p* < 0.001) respectively, whereas uISVC significantly deteriorated *WMP* by 14.26% ± 3.86% (*p* = 0.022).
(2) According to *WMP*, sPAC had superiority over uPAC by 21.48% ± 3.27% (*p* < 0.001), and sISVC had superiority over uISVC by 49.79% ± 5.48% (*p* < 0.001).
(3) According to *WMP*, uPAC had superiority over uISVC by 28.71% ± 5.66% (*p* = 0.001), whereas the recognition accuracy of sPAC had no significant difference with that of sISVC (*p* = 1).

## 4. Discussion

Interfering factors will cause concept drift of long term EMG signals. Experiments with long term EMG signals indicate that, when applying non-adapting PR-based methods to achieve dexterous and intuitive myoelectric control, the concept drift will lead to performance degradation. To solve the problem, we can use both supervised training samples and unsupervised newly labeled samples to update the classifier for tracking the concept drift. We proposed a particle adapting classifier (PAC), and compared it with incremental SVC (ISVC) and non-adapting SVC (NSVC) from both computational cost aspect and classifying performance aspect. The comparisons of adaptive classifiers (PAC and ISVC) were conducted in both supervised adaptive learning (noted as sPAC and sISVC) and unsupervised adaptive learning (noted as sPAC and sISVC) scenarios. To compare the classifying performance, we employed both simulated data sequence with adjustable concept drift (adjustable drift extent and adjustable drift rate), and realistic data from one-day-long acquiring experiments to imitate ordinary clinical use.

From computational cost aspect (Section 3.1), the proposed PAC overwhelmingly outperformed ISVC. The time cost per updating cycle of PAC, no matter in supervised or unsupervised adaptive learning scenarios, maintained less than 2 ms, whereas that of ISVC increased along with retraining times and reached almost 50 ms after 90,000 times of updating. One reason for the increasing time cost of ISVC was the increasing number of support vectors, as shown in Figure 11.

In order to compare the classification performance on realistic one-day-long data, we firstly plotted the changing tendency of recognition accuracy, and then defined the measures of *AER* and *WMP* to describe the performance diversity among classifiers. The value of *AER* was defined as the average overall recognition accuracy at the end of the day, whereas the value of *WMP* was defined as the average lowest motion recognition accuracy at the end of the day. According to the realistic one-day-long data, (Part 3.3) we could reach the following conclusions:

(1) At the beginning of the day, sISVC was with highest overall recognition accuracy at more than 96.02%. The other classifiers shared the similar overall recognition accuracy at around 85%. With time moving forward, sISVC and uPAC almost maintained the overall recognition accuracy at 92.67% and 83.93% respectively; sPAC improved the overall recognition accuracy to 91.23%; uISVC and NSVC deteriorated the overall recognition accuracy to 70.55% and 74.90% respectively. The overall recognition accuracy at the end of the day was measured by *AER*.
(2) According to the changing tendency within the day, supervised adaptive learning scenarios had superiorities on the performance over unsupervised adaptive learning scenarios (sISVC, sPAC, vs. uISVC, uPAC), while PAC had superiorities over ISVC (sPAC, uPAC, vs. sISVC, uISVC). It was remarkable that, in unsupervised adaptive learning scenarios, uPAC significantly improved the performance from NSVC, no matter for overall recognition accuracy *AER* (9.03% ± 2.23%, *p* = 0.011) or lowest motion recognition accuracy *WMP* (14.45% ± 4.06%, *p* = 0.028), whereas uISVC did not significantly improved the performance.
(3) The accuracy variations within one day for different motion types were different. Compared with wrist motion classifications, finger motion classifications had lower recognition accuracy. Among all motion types, Motion TF (thumb-index flexion) had the lowest classification recognition accuracy compared with other motion types. According to the confusion matrices of different motions, the confusion between Motion AF (all finger flexion) and Motion TF was the largest component of misclassification. Therefore, in future research, it is necessary to strengthen or compensate the classification of finger flexion movements.
(4) As shown in Figure 14 and Figure 16, the accuracy variation of NSVC shows continuous decreasing tendency on average, which implies the constantly increasing drift extent of the concept drift in the session sequence. Such session sequence would have similar property with the simulated data sequence.

**Figure 19:**
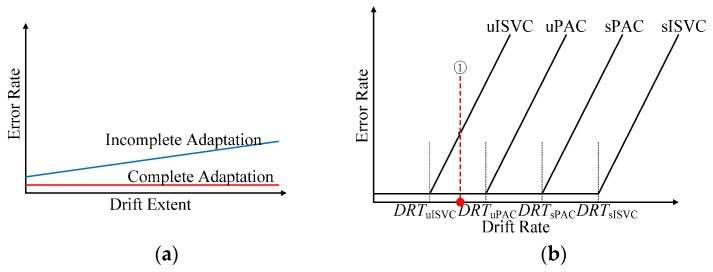
Schematic drawing of error rates changing along with drift extent and drift rate increasing. (**a**) Error rate changing along with drift extent increasing for complete adaption and incomplete adaption; (**b**) Error rate changing along with drift rate increasing, under the condition of fixed maximum drift extent. The plots of complete adaption and incomplete adaption in (**a**) are simplified from Figure 10. The plots in (**b**) are simplified from Figure 13. There are critical drift rates for adaptive classifiers, noted as *DRT*_u__ISVC_, *DRT*_uPAC_, *DRT*_sPAC_, *DRT*_s__ISVC_ respectively. When drift rate is smaller than the critical drift rate, the classifier can achieve complete adaption and keep the error rate low, when drift rate is larger than the critical drift rate, the adaption is incomplete adaption and the error rate increase dramatically along with drift rate increasing. (① the possible average drift rate of realistic data from one-day-long EMG acquiring trials).

With the simulated data sequence, we investigated how performance degradation was affected by the drift extent and the drift rate. Figure 19a illustrates the changing tendency of classifying error rates of sessions with fixed drift rate and increasing drift extent, while Figure 19b illustrates the changing tendency of classifying error rates of session sequences with fixed maximum drift extent and increasing drift rate. The figures are abstracted from Figure 12 and Figure 13. With simulated data, we could reach the following conclusions:

(a) No matter in supervised or unsupervised adaptive learning scenarios, the performance could be divided into two categories: *complete adaption*, the accuracy not falling down with drift extent increasing, and *incomplete adaption*, the accuracy falling down with drift extent increasing.
(b) There was a threshold of the drift rate for a particular adaptive strategy, noting as *DRT*. When and only when the drift rate of the dataset was less than *DRT*, the adaptive strategy achieved complete adaption. Therefore, *DRT* could be seen as the inherent property of an adaptive classifier. According to Figure 13, *DRT*_uISVC_ < *DRT*_uPAC_ < *DRT*_sPAC_ < *DRT*_sISVC_.
(c) When comparing the performances of NSVC in Figure 12 and Figure 14, we could find out that the average error rate of the realistic data sequence changed linearly within the day, showing the similarity to a simulated data sequence with a fixed drift rate between *DRT*_uPAC_ and *DRT*_uISVC_. The similarity could explain why uPAC, uISVC and sPAC maintained and even improved the recognition accuracy whereas uISVC deteriorated the recognition accuracy with time moving forward. Therefore, in the future, we could employ the drift rate threshold to describe the essential adaptability of an adaptive learning strategy.In this paper, we compared the performance of proposed PAC with incremental SVC and nonadapting SVC in both supervised and unsupervised adaptive learning scenarios. Compared with other classifiers, proposed PAC had two advantages: (a) the stable and small time cost per updating cycle, and (b) the capability to maintain or improve the classification performance no matter in supervised or unsupervised adaptive learning scenarios. The two advantages will contribute to performance improvement from conventional PR-based myoelectric control method in clinical use.

However, present experiments were based on offline data with algorithm deployed on a high-end PC platform, which could not simulate the realistic clinical usage perfectly. Therefore, in our future work, we will focus on the processing of online data with low-power and resource-constrained platform to investigate the realistic performance of our method.

## 5. Conclusions

We proposed a particle adaptive strategy to reduce the performance degradation in the long-term usage of myoelectric control with low computational cost. With validations of realistic one-day-long data, we concluded that the proposed PAC method had much lower computational cost than incremental SVC (time cost per updating cycle: 2 ms vs. 50 ms), and had better classification results (13.38% ± 2.62%, *p* = 0.001) in unsupervised adaptive learning scenarios and equivalent results (*p* = 1) in supervised adaptive learning scenarios compared with ISVC. At the same time, the proposed PAC method reduced the performance degradation of conventional non-adapting SVC no matter in supervised adaptive learning scenarios (16.32% ± 2.26%, *p* < 0.001) or unsupervised adaptive learning scenarios (9.03% ± 2.23%, *p* = 0.011). These results showed the great potential of employing the proposed method in myoelectric controlled prosthetic hands to improve the daily life of amputees.
